# UBA6 Inhibition Accelerates Lysosomal TRPML1 Depletion and Exosomal Secretion in Lung Cancer Cells

**DOI:** 10.3390/ijms25052843

**Published:** 2024-02-29

**Authors:** Dongun Lee, Peter Chang-Whan Lee, Jeong Hee Hong

**Affiliations:** 1 Department of Health Sciences and Technology, Lee Gil Ya Cancer and Diabetes Institute, GAIHST, Gachon University, 155 Getbeolro, Yeonsu-gu, Incheon 21999, Republic of Korea; sppotato1@gmail.com; 2 Department of Biomedical Sciences, University of Ulsan College of Medicine, Asan Medical Center, Seoul 05505, Republic of Korea; pclee@amc.seoul.kr

**Keywords:** UBA6, exosome, lung cancer, endosomal trafficking, multivesicular body

## Abstract

Ubiquitin-like modifier-activating enzyme 6 (UBA6) is a member of the E1 enzyme family, which initiates the ubiquitin–proteasome system (UPS). The UPS plays critical roles not only in protein degradation but also in various cellular functions, including neuronal signaling, myocardial remodeling, immune cell differentiation, and cancer development. However, the specific role of UBA6 in cellular functions is not fully elucidated in comparison with the roles of the UPS. It has been known that the E1 enzyme is associated with the motility of cancer cells. In this study, we verified the physiological roles of UBA6 in lung cancer cells through gene-silencing siRNA targeting *UBA6* (siUBA6). The siUBA6 treatment attenuated the migration of H1975 cells, along with a decrease in lysosomal Ca^2+^ release. While autophagosomal proteins remained unchanged, lysosomal proteins, including TRPML1 and TPC2, were decreased in siUBA6-transfected cells. Moreover, siUBA6 induced the production of multivesicular bodies (MVBs), accompanied by an increase in MVB markers in siUBA6-transfected H1975 cells. Additionally, the expression of the exosomal marker CD63 and extracellular vesicles was increased by siUBA6 treatment. Our findings suggest that knock-down of *UBA6* induces lysosomal TRPML1 depletion and inhibits endosomal trafficking to lysosome, and subsequently, leads to the accumulation of MVBs and enhanced exosomal secretion in lung cancer cells.

## 1. Introduction

Ubiquitination constitutes a pivotal cellular process orchestrating various cellular functions, including the ubiquitin–proteasome system (UPS), selective autophagy, cell signaling, endosomal trafficking, cell cycle, and cell death [1]. A deficiency in ubiquitin signaling induces cellular dysfunction and diseases, including aging, immune disorders, metabolic disorders, and cancer [1]. Target proteins are ubiquitinated through the binding of ubiquitin proteins [1]. In the ubiquitin cascade, ubiquitin-activating enzymes with E1, E2, and E3 series sequentially append ubiquitin to target substrate proteins [2].

UBA6 is known as an alternative E1 enzyme to UBA1 [3,4]. It has been known that UBA6 is associated with UBA6-specific E2-conjugating enzyme 1 (USE1) and reveals a specific binding affinity with USE1 [5]. Despite the identification of UBA6 and its widespread expression across various organs [5,6], information and experimental evidence regarding the role of UBA6 remain limited due to genetic phenotypes, such as embryonic lethality [7]. However, brain-specific *UBA6*-null mice have been generated and revealed impairment of the neuronal and behavioral phenotypes [7,8]. More recently, an up-regulation of UBA6 expression has been observed in stimulated T cells and involved in interferon-gamma production [9]. Although there was indirect evidence, enhanced expression of USE1 was revealed in lung cancer and its mutation was involved in tumor formation [10]. Moreover, the degradation cycle of the regulators of G protein signaling (RGS) proteins has been associated with the UBA6–USE1 cascade in vitro [11]. It has been known that UBA6 and the ubiquitin receptor complex are associated with USE1 [11].

The UPS regulates various cellular functions in the nervous system, cardiovascular system, and cancer [2]. In addition, the UPS plays roles in protein homeostasis, including stability and localization [2]. Similarly, lysosome-induced autophagy maintains protein homeostasis [12,13]. A recent study demonstrated that the UPS and the autophagy interact with each other to mutually supplement their functions [14]. Thus, we speculated that the evaluation of changes in lysosome activity is considered a tool for the verification of roles in ubiquitination.

The lysosomes are activated by lysosomal Ca^2+^ ([Ca^2+^]_Lys_) [15]. The [Ca^2+^]_Lys_ release is associated with the modulation of cellular migration [16]. The transient receptor potential mucolipin 1 (TRPML1) is the lysosomal calcium channel, which is involved in cellular migration [17]. Enhanced invasive property was revealed in USE1-overexpressed lung cancer cells [11]. In contrast, *UBA1* depletion decreased cellular proliferation, migration, and invasion in the hepatoma-derived Huh7 cell line [18]. We hypothesized that UBA6 possesses a modulatory effect on cellular motility. Thus, we determined the regulatory role of UBA6 in cellular motility. The modulatory mechanism of UBA6 in cellular migration was determined in lung cancer cells, and the cellular responses were evaluated in *UBA6* depletion.

## 2. Results

### 2.1. Knockdown of UBA6 Attenuated Lung Cancer Cell Migration and [Ca^2+^]_Lys_ Release

**Figure 1:**
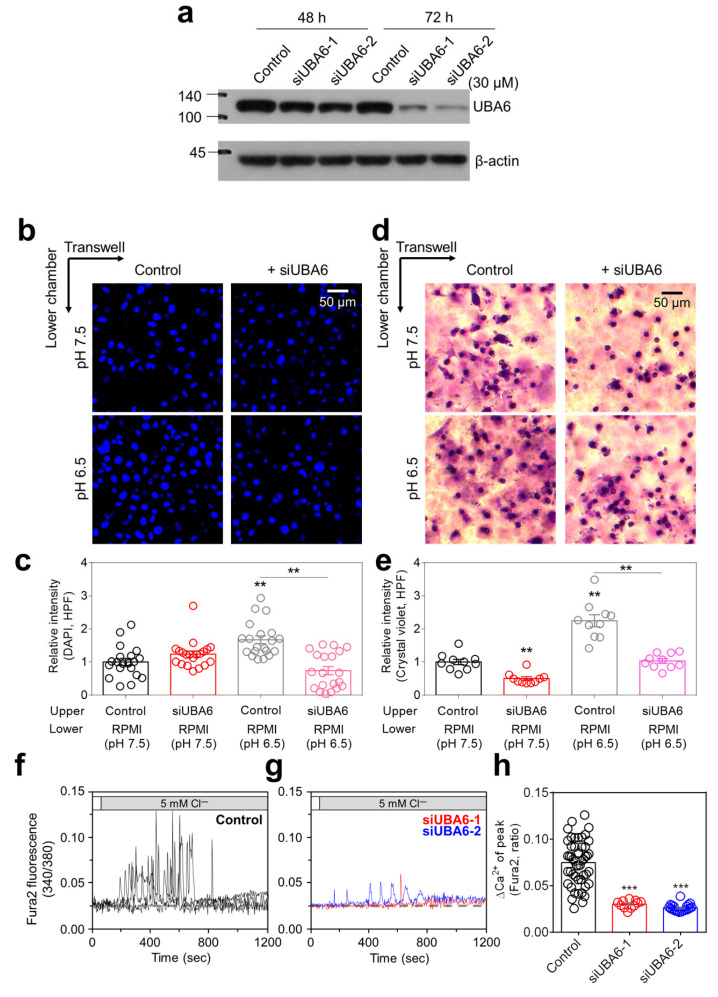
Inhibitory role of siUBA6 in H1975 cells. (**a**) Western blotting analysis of UBA6, which is transfected with siControl (Control) and siUBA6 to evaluate the efficacy of siUBA6. β-actin was used as a loading control. (**b**) Images of the transwell migration assay with DAPI, which is incubated with siUBA6 in H1975 cells. The scale bar represents 50 μm. (**c**) The dot plots are presented as the means ± SEMs of the relative intensity of DAPI (n = 20, ** *p* < 0.01). (**d**) Images of the transwell migration assay with crystal violet, which is incubated with siUBA6 in H1975 cells. The scale bar represents 50 μm. (**e**) The dot plots are presented as the means ± SEMs of the relative intensity of crystal violet (n = 10, ** *p* < 0.01). (**f**,**g**) 5Cl^−^-induced Ca^2+^ signaling in H1975 cells with Control (**f**) and transfection of siUBA6-1/siUBA6-2 (**g**) for 72 h. (**h**) The dot plots are presented as the means ± SEMs of the relative changes in Ca^2+^ spikes (ΔCa^2+^, n = 49, n = 11, n = 21, *** *p* < 0.001).

To evaluate the physiological mechanism of UBA6, small interfering RNA (siRNA) targeting UBA6 (siUBA6) was used in H1975 lung cancer cells. Both types of siUBA6 decreased UBA6 expression in H1975 cells treated for 72 h (Figure 1a). For subsequent experiments, the siUBA6-2 was mainly used in this study. It has been known that the migration of lung cancer cells is increased in an acidic environment [19]. H1975 cell migration was enhanced in pH 6.5 media, whereas knockdown of UBA6 attenuated pH 6.5 media-induced H1975 migration (Figure 1b–e). To verify the role of siUBA6 in other cancer cell lines, H1299 (p53-null type, invasive cells) and A549 (wild-type p53, noninvasive cells) were used. The cellular viability and apoptotic reaction of lung cancer cells, including H1975, H1299, and A549, showed no significant changes by siUBA6 (Supplementary Figure S1). [Ca^2+^]_Lys_ release, known to induce autophagic flux, is implicated in promoting cancer cell migration [16,20,21]. Our previous study showed a method utilizing the low Cl^−^ to mediate [Ca^2+^]_Lys_ release [22]. Thus, we investigated whether the inhibition of UBA6 induces [Ca^2+^]_Lys_ in H1975 cells. The 5 mM Cl^−^ solution induced intracellular Ca^2+^ signaling in H1975 cells (Figure 1f). However, knockdown of UBA6 by siUBA6 attenuated low Cl^−^-induced Ca^2+^ signaling in H1975 cells (Figure 1g,h). These results indicate that down-regulation of UBA6 reduces lung cancer cell migration and [Ca^2+^]_Lys_ release.

### 2.2. Knockdown of UBA6 Attenuated TRPML1-Mediated [Ca^2+^]_Lys_ Release

**Figure 2:**
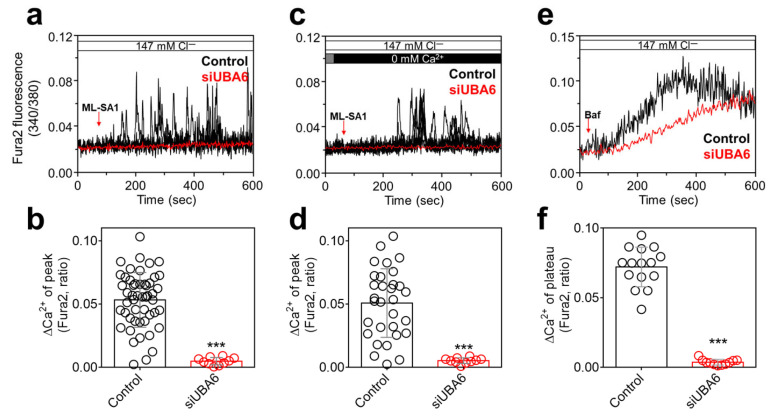
Inhibitory role of siUBA6 in TRPML1-induced [Ca^2+^]_Lys_ release. (**a**) Changes in Ca^2+^ signaling in siUBA6-transfected H1975 cells with the treatment of ML-SA1 in the presence of Ca^2+^. (**b**) The dot plots are presented as the means ± SEMs of the relative changes in Ca^2+^ plateau or spikes (ΔCa^2+^, n = 50, n = 10, *** *p* < 0.001). (**c**) Changes in Ca^2+^ signaling in siUBA6-transfected H1975 cells with the treatment of ML-SA1 in the absence of Ca^2+^. (**d**) The dot plots are presented as the means ± SEMs of the relative changes in ΔCa^2+^ (n = 31, n = 10, *** *p* < 0.001). (**e**) Changes in Ca^2+^ signaling in siUBA6-transfected H1975 cells with the treatment of Baf. (**f**) The dot plots are presented as the means ± SEMs of the relative changes in ΔCa^2+^ (n = 16, n = 11, *** *p* < 0.001).

The [Ca^2+^]_Lys_ release is mainly induced by the transient receptor potential (TRP) mucolipin (TRPML)1 [15,23]. We evaluated whether siUBA6 inhibited [Ca^2+^]_Lys_ release through the inhibition of TRPML1. To activate TRPML, the TRPML activator ML-SA1 [24] was used. Knockdown of *UBA6* attenuated ML-SA1-induced [Ca^2+^]_Lys_ release in the presence or absence of extracellular Ca^2+^ (Figure 2a–d). To confirm the inhibitory effect of siUBA6 on [Ca^2+^]_Lys_ release, the [Ca^2+^]_Lys_-releasing reagent, bafilomycin A1 (Baf) [25], was treated in H1975 cells. The Baf-induced Ca^2+^ response was decreased by siUBA6 (Figure 2e,f). These results indicate that the inhibition of *UBA6* attenuates TRPML1-mediated [Ca^2+^]_Lys_ release.

### 2.3. Knockdown of UBA6 Attenuated TRPML1 Expression

**Figure 3:**
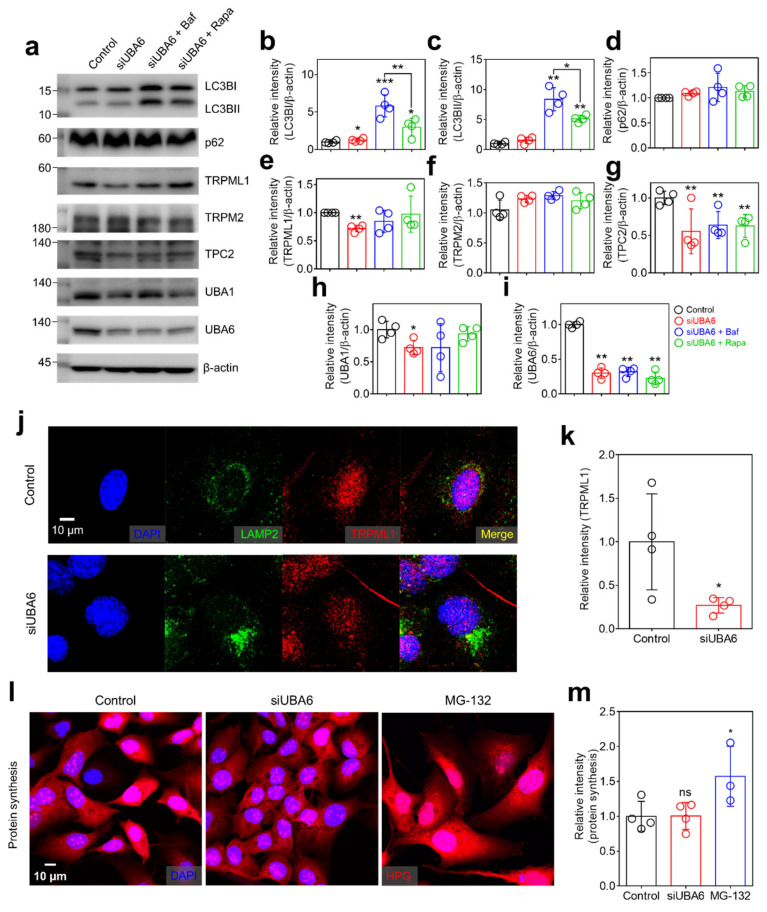
The effect of siUBA6 in the lysosomal complex. (**a**) Western blotting analysis of LC3B, p62, TRPML1, TRPM2, TPC2, UBA6, and UBA1 transfected with siUBA6 in the presence or absence of Baf and rapamycin. β-actin was used as a loading control. (**b**–**i**) The dot plots are presented as the means ± SEMs of the protein bands normalized to β-actin (n = 4, * *p* < 0.05, ** *p* < 0.01, *** *p* < 0.001). (**j**) Images of immunofluorescence staining of LAMP2 (green), TRPML1 (red), and DAPI (blue) in siUBA6-transfected H1975 cells. The scale bar represents 10 μm. (**k**) The dot plots are presented as the means ± SEMs of the relative intensity of TRPML1 (n = 4, * *p* < 0.05). (**l**) Images of HPG in siUBA6-transfected H1975 cells (red) with DAPI (blue). The scale bar represents 10 μm. (**m**) The dot plots are presented as the means ± SEMs of the relative intensity of HPG (n = 4, ns = non-significant, * *p* < 0.05).

Lysosomes play a critical role in the autophagic flux [26]. The inhibition of the UPS induces the compensatory activation of autophagy and degradation of proteins [27,28]. To demonstrate whether modulation of *UBA6* is related to the autophagic flux, the effect of siUBA6 on the autophagic and lysosomal proteins was verified. The expression of LC3BI was slightly increased, whereas the components of autophagosomes, LC3BII and p62 [29], remained unchanged by siUBA6 treatment (Figure 3a–d). The autophagy inhibitor Baf [30] and autophagy inducer rapamycin (Rapa [31]) increased LC3B expression without a change in p62 expression (Figure 3a–d). The increase in LC3B expression was higher in the Baf treatment than in the Rapa treatment (Figure 3a–c). TRPML1 was decreased by siUBA6 treatment (Figure 3a,e). Plasma membrane-associated TRP melastatin 2 (TRPM2) was not changed in the presence of siUBA6 (Figure 3a,f). Additionally, another Ca^2+^ channel in lysosome, TPC2 [32,33,34], was decreased by siUBA6 (Figure 3a,g). Knockdown of *UBA6* mildly down-regulated UBA1 expression (Figure 3a,h). The expression of UBA6 was verified with the indicated conditions (Figure 3a,i). To verify the reduced [Ca^2+^]_Lys_ release in the presence of siUBA6, we determined the cellular localization of TRPML1 and TPCs. SiUBA6-treated cells exhibited reduced TRPML1 intensity (Figure 3j,k). Lysosome-associated membrane protein 2 (LAMP2), a lysosomal marker [35], aggregated in siUBA6-treated cells (Figure 3j,k). To confirm the effect of siUBA6 on the proteolysis pathway, the degradation of homopropargylglycine (HPG) was measured. The intensity of Alexa 594-conjugated HPG was not changed by siUBA6 compared to the control (Figure 3l,m). Otherwise, the proteasome inhibitor MG-132 [36] treatment increased the HPG intensity (Figure 3l,m), suggesting that inhibition of UBA6 increases the autophagic flux to eliminate siUBA6-mediated non-degraded proteins. Our previous study demonstrated that the excessive autophagy through low Cl^−^ induced lysosomal depletion [22]. These results demonstrate that siUBA6-induced inhibition of [Ca^2+^]_Lys_ release is derived from the down-regulation of TRPML1 expression with the increase in autophagy.

### 2.4. Knockdown of UBA6 Mediated Intracellular Vesicle Trafficking through Multivesicular Body (MVB)

**Figure 4:**
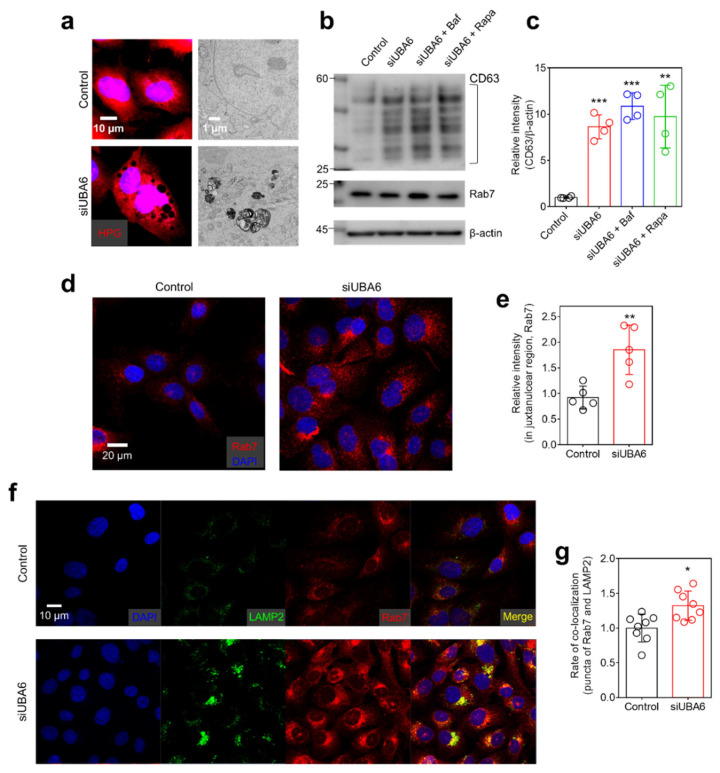
The effect of siUBA6 in MVBs. (**a**) Left panel: Images of HPG in siUBA6-transfected H1975 cells (red) with DAPI (blue). The scale bar represents 10 μm. Right panel: Transmission electron microscopy images of UBA6-depleted H1975 cells with siUBA6 treatment. The scale bar represents 1 μm. (**b**) Western blotting analysis of CD63 and Rab7, which are transfected with siUBA6 in the presence or absence of Baf and rapamycin. β-actin was used as a loading control. (**c**) The dot plots are presented as the means ± SEMs of the relative intensity of CD63 (n = 4, ** *p* < 0.01, *** *p* < 0.001). (**d**) Images of immunofluorescence staining of Rab7 (red) and DAPI (blue) in siUBA6-transfected H1975 cells. The scale bar represents 20 μm. (**e**) The dot plots are presented as the means ± SEMs of the relative intensity of CD63 (n = 5, ** *p* < 0.01). (**f**) Images of immunofluorescence staining of LAMP2 (green), Rab7 (red), and DAPI (blue) in siUBA6-transfected H1975 cells. The scale bar represents 10 μm. (**g**) The dot plots are presented as the means ± SEMs of the co-localization rate of Rab7 and LAMP2 (n = 9, * *p* < 0.05).

Numerous puncta were observed in siUBA6-transfected cells and magnified HPG images revealed enlarged vesicles (Figure 4a, left side). Transmission electron microscopy (TEM) showed an increased number of enlarged intracellular vesicles in siUBA6-transfected cells (Figure 4a, right side). Thus, we hypothesized that the knockdown of *UBA6* is associated with the enlargement of MVBs. To confirm whether siUBA6 increases MVBs, the expression of CD63 and Rab7 (MVB markers) [37,38] was measured. Knockdown of *UBA6* induced the increased expression of CD63 in H1975 and other lung cancer cells, including H1299 and A549 (Figure 4b,c and Figure S2). Autophagic modulation through Baf and Rapa had no effect on the siUBA6-induced CD63 increase (Figure 4b,c). Although the expression of Rab7 was not changed (Figure 4b), the translocation of Rab7 to the juxtanuclear region was increased by the siUBA6 treatment (Figure 4d,e). To verify lysosomal modulation by siUBA6, the expression of the lysosomal marker LAMP2 was determined in siUBA6-transfected cells. As shown in Figure 3j,k, siUBA6 increased LAMP2 repositioning to the juxtanuclear region in spite of the lysosomal depletion. Lysosomes fuse with MVBs to release exosomes, which is mediated by LAMP2 [39]. Thus, we analyzed the relationship between MVBs and lysosomes. The MVB marker Rab7 and lysosomal marker LAMP2 were co-localized in siUBA6-transfected cells. (Figure 4f,g). These results indicate that the knockdown of *UBA6* modulates intracellular vesicle trafficking through the enlargement of MVBs, with an increase of CD63 expression and Rab7 repositioning to the juxtanuclear region.

### 2.5. Knockdown of UBA6 Induced Exosomal Secretion

**Figure 5:**
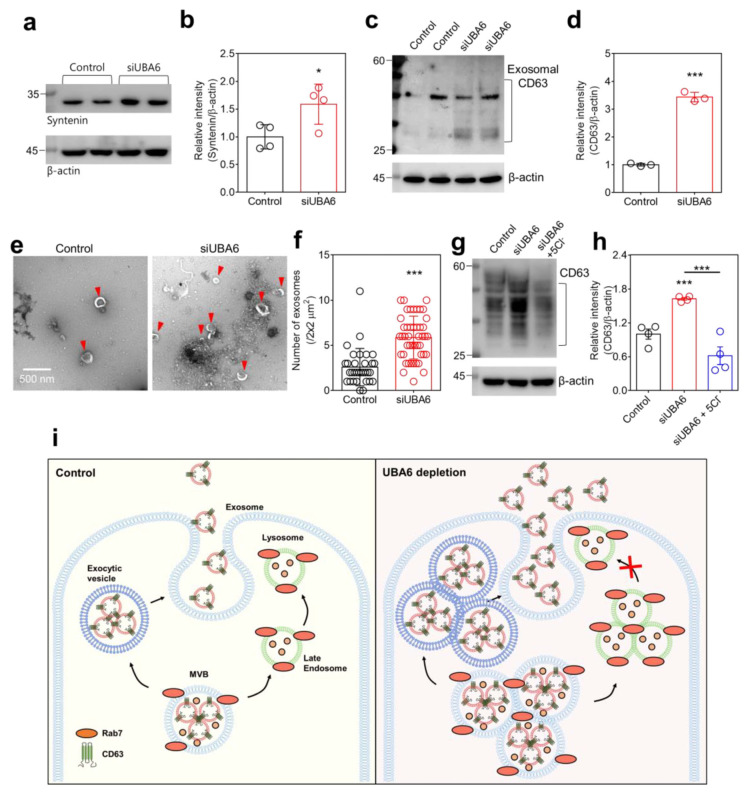
The increase in exosomal secretion through UBA6 depletion. (**a**) Western blotting analysis of syntenin, where β-actin was used as a loading control. (**b**) The dot plots are presented as the means ± SEMs of the protein band of syntenin normalized to β-actin (n = 4, * *p* < 0.05). (**c**) Western blotting analysis of CD63, which is transfected with siUBA6 in isolate exosomes. β-actin was used as a loading control. (**d**) The dot plots are presented as the means ± SEMs of the protein band of CD63 normalized to β-actin (n = 3, *** *p* < 0.001). (**e**) Transmission electron microscopy images of exosomes (red arrow heads) in H1975-cultured media with siUBA6. The scale bar represents 500 nm. (**f**) The dot plots are presented as the means ± SEMs of a number of exosomes (n = 32, n = 46, *** *p* < 0.001). (**g**) Western blotting analysis of CD63 from the total cell extraction, where β-actin was used as a loading control. (**h**) The dot plots are presented as the means ± SEMs of the protein band of CD63 normalized to β-actin (n = 4, *** *p* < 0.001). (**i**) Schematic illustration of the mechanism of UBA6-mediated endosomal-/exosomal-trafficking by increasing MVBs. Small pink-colored dots represent degradable proteins.

Lysosomal degradation of MVBs through phosphate and the tensin homolog (PTEN) reduces the secretion of exosomes, whereas inhibition of lysosomal activity causes the opposite effect [40]. Exosomes, responsible for transporting various molecules, are marked by the expression of CD proteins, including CD63 [41]. Exosomal secretion is controlled by syntenin, which interacts with CD63 [42]. The increase in syntenin expression accompanies the increase in CD63 expression [42]. Thus, we hypothesized that knockdown of *UBA6* induces exosomal secretion, along with an increase in MVB and CD63 expression. We confirmed that the siUBA6 increased the total expression of syntenin in H1975 cells (Figure 5a,b). To evaluate whether the siUBA6-induced increase in MVB and CD63 involves exosomal secretion, secreted exosomes were isolated from H1975 cell culture media. The knockdown of *UBA6* increased the exosomal CD63 in H1975 cells (Figure 5c,d). In addition, an increased size and number of exosomes were observed in cell-cultured media in the presence of siUBA6 (Figure 5e,f). In a previous study, we demonstrated that the low Cl^−^ induces lysosomal depletion [22]. The increased total expression of CD63 by the siUBA6 treatment was attenuated by treatment with 5 mM Cl^−^ solution in H1975 cells (Figure 5g,h). These results demonstrate that the knockdown of *UBA6* treatment increased MVBs, subsequently inducing exosomal secretion, as illustrated in Figure 5i.

## 3. Discussion

In this study, *UBA6* depletion by siRNA attenuated cellular migration and induced the depletion of TRPML1-mediated [Ca^2+^]_Lys_ release. Knockdown of *UBA6* decreased the expression of lysosomal proteins, including the Ca^2+^ channels TRPML1 and TPC2. Although knockdown of *UBA6* inhibited both proteolytic processes, the UPS and lysosomal activity, the total protein level was sustained. *UBA6* depletion increased MVBs, marked by Rab7 and CD63, and induced exosomal secretion. The increased CD63 through siUBA6 was attenuated by treatment with 5 mM Cl^−^ solution-induced lysosome depletion.

TRPML1 is a major channel component of lysosomes used to regulate lysosomal functions and repositioning by transporting [Ca^2+^]_Lys_ to the cytosol [43,44]. Although the TRPMLs mainly operate and express in lysosomes, these channels move from the plasma membrane to lysosomes through endosomal trafficking [45,46]. Endosomal trafficking is preceded by [Ca^2+^]_Lys_ signaling, and the activation of TRPML1-induced Ca^2+^ signaling has a relationship with MVBs. [Ca^2+^]_Lys_ release through TRPML1 triggers lysosomal trafficking to fuse with MVBs and to degrade MVBs, which finally decreases inflammatory exosomal secretion [47]. Deletion of *TRPML1* genes increases extracellular vesicles and CD63 expression in the mouse artery wall [48]. It is known that the CD63 is essential for intracellular trafficking and serves as an exosomal marker [49,50]. Thus, the modulation of ubiquitin through UBA6 can be suggested as a potential therapeutic target for vesicle trafficking-related diseases [51]. Moreover, proteins in extracellular vesicles are glycosylated in various regions with various types of glycosylation [52,53]. Understanding the glycosylation patterns of cancer extracellular vesicles is suggested as a therapeutic strategy for cancer [54]. The glycosylation of CD63 was quantified through Western blot analysis, revealing a spectrum of bands ranging in size from 30 to 70 kDa [55]. Although the specific types of glycosylation were not verified, the bands of around 30 kDa was increased, while the bands at 45 kDa exhibited no significant alterations in siUBA6-transfected cells (Figure 5c).

Ubiquitination is necessary for the proteolytic pathways, including not only the UPS but also autophagy [56]. Misfolded proteins are ubiquitinated and subsequently pass through the UPS for degradation [56]. However, ubiquitinated aggregates binds with p62 to form inclusion bodies, and then the p62 with ubiquitinated aggregates binds with LC3B in lysosomes for degradation through autophagy [56]. In addition, ubiquitination is necessary for endosomal trafficking to deliver plasma membrane proteins to lysosomes [57]. Although there is ongoing debate regarding the ubiquitination of several proteins in exosomes [58], the ubiquitination status determines whether the proteins pass through lysosomal trafficking or exosomal secretion. Ubiquitinated proteins transport to MVBs and bind with the endosomal sorting complex required for transport (ESCRT)-0,I,II for delivery to lysosomes [59]. Conversely, non-ubiquitinated proteins bind the syndecan/syntenin complex for exocytosis through exosomes [59]. Thus, the inhibition of ubiquitination has the potential to inhibit endosomal trafficking to lysosome and to trigger exosomal secretion.

In this study, the knockdown of *UBA6* triggered exosomal secretion through inhibition of endo-lysosomal trafficking. The deficiency in endo-lysosomal trafficking reduced the expression of TRPML1 and subsequently attenuated TRPML1-induced [Ca^2+^]_lys_ signaling. In addition, the decreased [Ca^2+^]_lys_ release was involved in the attenuated migration of lung cancer cells. It thus appears that inhibited ubiquitination through *UBA6* deficiency possesses potential as a therapeutic approach for targeting endo-lysosomal trafficking in lung cancer.

## 4. Materials and Methods

### 4.1. Reagents and Cell Culture

Fura-2-AM (Fura-2) was purchased from TEFlabs (Austin, TX, USA; Cat: 0102). Pluronic acid (Pluronic F-127, 20%(*v*/*v*) in DMSO) was purchased from Invitrogen (Carlsbad, CA, USA; Cat: P-3000MP). Baf (from *Streptomyces griseus*) and ML-SA1 were purchased from Sigma (St Louis, MO, USA; Cat: B1793 and SML0627). Non-small-cell lung cancer cell lines H1975, H1299, and A549 were obtained from the American Type Culture Collection (Rockville, MD, USA; Cat: CRL-5908, CRL-5803, and CCL-185). H1975 and H1299 cells were maintained in Roswell Park Memorial Institute 1640 (RPMI 1640, Invitrogen, Cat: 11875-093) containing 10% fetal bovine serum (FBS, Invitrogen, Waltham, MA, USA; Cat: 16000-044) and 100 U/mL penicillin–streptomycin (Invitrogen, Cat: 15140122). A549 cells were maintained in Dulbecco’s modified Eagle’s medium (Invitrogen, Cat: 11995-065) containing 10% fetal bovine serum and 100 U/mL penicillin–streptomycin. The cells were incubated in a humidified incubator with 5% CO_2_ and 95% air at 37 °C. When the cells reached 80% confluence, they were washed with Dulbecco’s phosphate-buffered saline (Welgene, Geyngsan, Republic of Korea; Cat: LB001-02) after the culture medium was removed. The cells were treated with trypsin/EDTA (Invitrogen; Cat: 25200-072) for 5 min. The detached cells were transferred to fresh culture dishes with or without coverslips for the measurement of migration assay, Western blotting, measurement of intracellular calcium ([Ca^2+^]_i_), confocal microscopy, detection of intracellular protein level, and transmission electron microscopy.

### 4.2. siRNA and Transfection

The siUBA6 were constructed by Genolution (Seoul, Republic of Korea), where the sequences were 5′-CAAAGUAACUGGUGGCUAU-3′ (siUBA6-1), and 5′-CCUUGGAAGAGAAGCCUGAUGUAAA-3′ (siUBA6-2). The siControl was non-targeting RNA, also constructed by Genolution. The siRNAs were transfected using Lipofectamine 2000 (Invitrogen; Cat: 11668019) according to the manufacturer’s protocol. Each siRNA was diluted in 200 μL of Opti-Eagle’s minimum essential media (Invitrogen, 31985-070) and mixed with Lipofectamine 2000 mixture. The final mixture was incubated for 10 min at room temperature (RT) and then transferred to H1975 cells with fresh RPMI media. After 6 h of incubation, the medium was replaced with fresh medium, and the cells were incubated for 72 h.

### 4.3. Transwell Membrane Migration Assay

The detached H1975 cells (5 × 10^4^ cells/well) were dispersed in RPMI (with 1% FBS) and transferred to the upper chamber of a 24-transwell membrane plate (8.0 μm pore-sized insert). The lower chambers were filled with pH 7.5- or pH 6.5-adjusted RPMI (with 10% FBS) with the indicated conditions. After incubation for 3 h, the membranes were stained with DAPI (blue) or crystal violet (purple). The membranes were incubated with chilled methanol (preserved at −20 °C) for 1 min at −20 °C to fix the cells, and then washed with PBS three times. For the DAPI staining, the membranes were incubated with DAPI solution (1 μg/mL in distilled water (DW)) for 30 min at 37 °C in the dark and then washed twice with DW. For the crystal violet staining, the membranes were incubated with 0.25% crystal violet solution in DW (with 20% methanol) for 10 min at room temperature in the dark, and then washed with DW for six times. After washing the membranes, the media on the top were carefully removed, and DW was added to the bottom of the plate. The plates were subsequently analyzed using an LSM 700 Zeiss confocal microscope (Carl Zeiss, Jena, Germany) (for DAPI) or an inverted microscope (Olympus, Tokyo, Japan) with Mosaic software (2.1 ver., Opto Science, Tokyo, Japan) (for crystal violet). The intensity of the obtained images was measured using the Meta Morph system (Molecular Devices, San Jose, CA, USA).

### 4.4. Western Blotting

The cells incubated with the indicated conditions were dispersed in lysis buffer (Cell Signaling, Danvers, MA, USA; Cat: 9803) containing 20 mM Tris, 150 mM NaCl, 2 mM EDTA, 1% Triton X-100, and protease inhibitor mixture. The cell lysate was sonicated and centrifuged at 11,000× *g* for 15 min at 4 °C. The protein concentration was calculated using the Bradford assay kit (Bio-Rad, Hercules, CA, USA; Cat: 5000001). The obtained protein samples were incubated with sodium dodecyl sulfate (SDS) protein sample buffer, and then the samples were separated by SDS-polyacrylamide gel electrophoresis, followed by transfer onto polyvinylidene difluoride membranes soaked in methanol. The membranes were incubated for 1 h at RT with 5% non-fat milk solution in 0.05% Tris-buffered saline with 0.05% Tween-20 to block non-specific binding. The blocked membranes were incubated overnight with the primary antibodies at 4 °C. UBA6, UBA1, TRPM2, CD63, Rab7, syntenin, and p62 were detected using the UBA6, UBA1, TRPM2, CD63, Rab7, syntenin, and sequestosome 1 (SQSTM1) antibodies (Abcam, Cambridge, UK; Cat: ab228918, ab181225, ab11168, ab134045, ab137029, ab133267, and ab56416, respectively). LC3B was detected using the LC3B antibody (Novusbio, Centennial, CO, USA; Cat: NB100-2220). TRPML1 was detected using the TRPML1 (Mucolipin 1) antibody (Alomone Labs, Jerusalem, Israel; Cat: ACC-081). TPC2 was detected using the TPCN2 antibody (Alomone Labs; Cat: ACC-072). After primary antibody incubation, the membranes were washed with PBS three times and then incubated with the secondary antibodies: horseradish peroxidase-conjugated mouse IgG and rabbit IgG antibodies (Millipore, Billerica, MA, USA; Cat: AP124P and AP132P) for 1 h at RT. β-actin was detected using a horseradish peroxidase-conjugated β-actin antibody (Sigma, Tokyo, Japan; Cat: A3854). The membrane was washed three times, and the protein was detected with enhanced chemiluminescence with the ImageQuant 800 (Cytiva) imaging system.

### 4.5. Measurement of [Ca^2+^]_i_ Concentration

The H1975 cells transferred onto coverslips were incubated with 4 μM Fura-2-AM and 0.05% pluronic acid in physiological salt solution (Reg) (containing 147 mM Cl^−^, Supplementary Figure S3) for 15 min at RT in the dark. After incubation with Fura-2, the cells were washed with Reg for 5 min before measuring the [Ca^2+^]_i_. The time course of the solutions applied was represented by bars above the traces. [Ca^2+^]_i_ was determined by measuring the Fura-2 fluorescence using dual excitation wavelengths of 340 nm and 380 nm and an emission wavelength of 530 nm. [Ca^2+^]_i_ was represented by the Fura-2 fluorescence ratio (340/380). The emitted fluorescence was monitored using a CCD camera (Retiga 6000, Q-imaging, Tucson, AZ, USA) attached to an inverted microscope (Olympus) and analyzed using a Meta Fluor system (Molecular Devices). The fluorescence images, obtained at 3 s intervals, were normalized by subtracting the raw signals from the background images.

### 4.6. Immunofluorescence and Confocal Microscopy

The H1975 cells transferred onto coverslips were incubated with the indicated conditions and then fixed with 4% paraformaldehyde (PFA) in PBS for 10 min at RT. The fixed cells were washed three times with PBS and then incubated with blocking serum (0.5% BSA and 5% goat serum in PBS) for 1 h at RT to block the non-specific binding sites. The blocked cells were incubated with the primary antibodies overnight at 4 °C, followed by three washes with PBS at RT. Lysosomal vesicles were stained with the LAMP2 antibody (1:100) (Abcam; Cat: ab25631), which was detected by incubation with the fluorescein isothiocyanate-tagged mouse IgG antibody (green, 1:200, Jackson ImmunoResearch, West Grove, PA, USA; Cat: 115-095-003) for 1 h at RT. TRPML1 was stained with the TRPML1 antibody (1:100), which was detected by the rhodamine-tagged mouse IgG antibody (red, 1:200, Jackson ImmunoResearch; Cat: 115-025-072) for 1 h at RT. Multi-vesicular bodies were stained with the CD63 and Rab7 antibodies (1:100), which were detected by incubation with the rhodamine-tagged rabbit IgG antibody (red, 1:200) for 1 h at RT. After incubation, the cells were washed three times, and the cover slips were mounted on a glass slide with 20 μL of Fluoromount-G containing 4′,6-diamidino-2-phenylindole (DAPI) (Electron Microscopy Sciences, Hatfield, PA, USA; Cat: 17984-24). Confocal microscopy images were obtained using an LSM 700 Zeiss confocal microscope (Carl Zeiss, Germany) with ZEN software (3.7, Carl Zeiss).

### 4.7. Detection of Intracellular Protein Level

The H1975 cells transferred onto coverslips were incubated with the indicated conditions and then labeled with Alexa Fluor 594 tagged-HPG using the Click-iT HPG Alexa Fluor Protein Synthesis Assay Kit (Invitrogen; Cat: C10429). As following the instructions, the cells were incubated with HPG working solution in L-methionine-free RPMI (Invitrogen; Cat: A1451701) for 30 min at 37 °C. After incubation, the cells were washed once with PBS. The washed cells were fixed with 4% PFA solution for 15 min at RT and then washed twice time with 3% BSA in PBS. The cover slips were mounted on slide glasses with 20 μL of Fluoromount-G containing DAPI (Electron Microscopy Sciences). The images were obtained using an LSM 700 Zeiss confocal microscope (Carl Zeiss) with ZEN software (3.7 Ver., Carl Zeiss). The intracellular protein level was calculated by the intensity of Alexa Fluor 594, relatively.

### 4.8. Analysis of Cellular Viability and Apoptosis

To analyze the cellular viability and apoptosis of H1975, H1299, and A549 cells, 3-(4,5-Dimethylthiazol-2-yl)-2,5-Diphenyltetrazolium Bromide) (MTT) assay and flow cytometry were performed. For the MTT assay, the cells were put onto a 96-well plate and then siUBA6 was transfected into the cells. After incubation for 72 h, 1 mg/mL of MTT dye (Sigma, Cat: 475989) was treated in the cells and incubated for 2 h in the dark. After 2 h, the incubated medium was carefully discarded and 100 μL of DMSO was added to the plates. The absorbance was measured at 570 nm using a fluorescence microplate reader (VARIOSCAN LUX, Thermo, Waltham, MA, USA). The percentage of bars was normalized to 100% by the control. For the flow cytometry, in brief, siUBA6-transfected cells were re-suspended in 100 μL annexin V binding buffer (50 mM HEPES, 700 mM NaCl, 12.5 mM CaCl_2_, pH 7.4). Thereafter, the cells were incubated with 5 μL of annexin V (Thermo, Cat: A35122) and 1 μL of propidium iodide (PI, 100 μg/mL, Thermo, Cat: P1304MP) at room temperature for 15 min. After incubation, 500 μL of annexin V buffer was added. The samples were analyzed at 410 nm excitation with a 455 nm band-pass filter to detect Pacific blue and 535 nm excitation with a 620 nm band-pass filter to detect PI.

### 4.9. Exosome Purification and Transmission Electron Microscopy

After incubation with siUBA6, the cultured media of H1975 cells were collected in tubes for the purification of the exosomes. The exosomes were isolated using the exoEasy Maxi kit (QIAGEN, Germantown, MD, USA, Cat: 76064), as following the manufacturer’s instructions. Briefly, the collected media were mixed with 1 volume of XBP buffer. The mixture was transferred to the spin column and centrifuged at 500× *g* for 1 min. XWP buffer was added to the spin column and centrifuged at 3000× *g* for 5 min. The spin columns were transferred to new tubes and XE buffer was added to elute the exosomes. The products were incubated with protein sample buffer at 95 °C for 5 min. The samples were used for Western blotting. For the TEM, the isolated exosomes were transferred onto a formvar/carbon-coated Ni. grid and then stained with 5% uranyl acetate for 3 min. The samples were washed and dried for 30 min. The images were obtained using a transmission electron microscope (JEM-1011, JEOL, Tokyo, Japan) at an acceleration voltage of 80 kV.

### 4.10. Statistical Analyses

Data from the indicated number of experiments were expressed as the mean ± standard error of the mean (SEM). Statistical significance was determined by analysis of variance for each experiment (* *p* < 0.05, ** *p* < 0.01, *** *p* < 0.001), which was analyzed using ANOVA (followed by a Newman–Keuls multiple comparison test).

## Data Availability

The data presented in this study are available on request from the corresponding author. The data are not publicly available.

## Supplementary Materials

The following supporting information can be downloaded at https://www.mdpi.com/article/10.3390/ijms25052843/s1.
