# Supplementary material for: UBA6 Inhibition Accelerates Lysosomal TRPML1 Depletion and Exosomal Secretion in Lung Cancer Cells
Source: Int J Mol Sci. 2024 Feb 29;25(5):2843. doi: 10.3390/ijms25052843 (PMC10932338; doi:10.3390/ijms25052843)
Supplement: Supplementary file 1 [file ijms-25-02843-s001.zip › ijms-2799498-supplementary.pptx]

## Slide 1
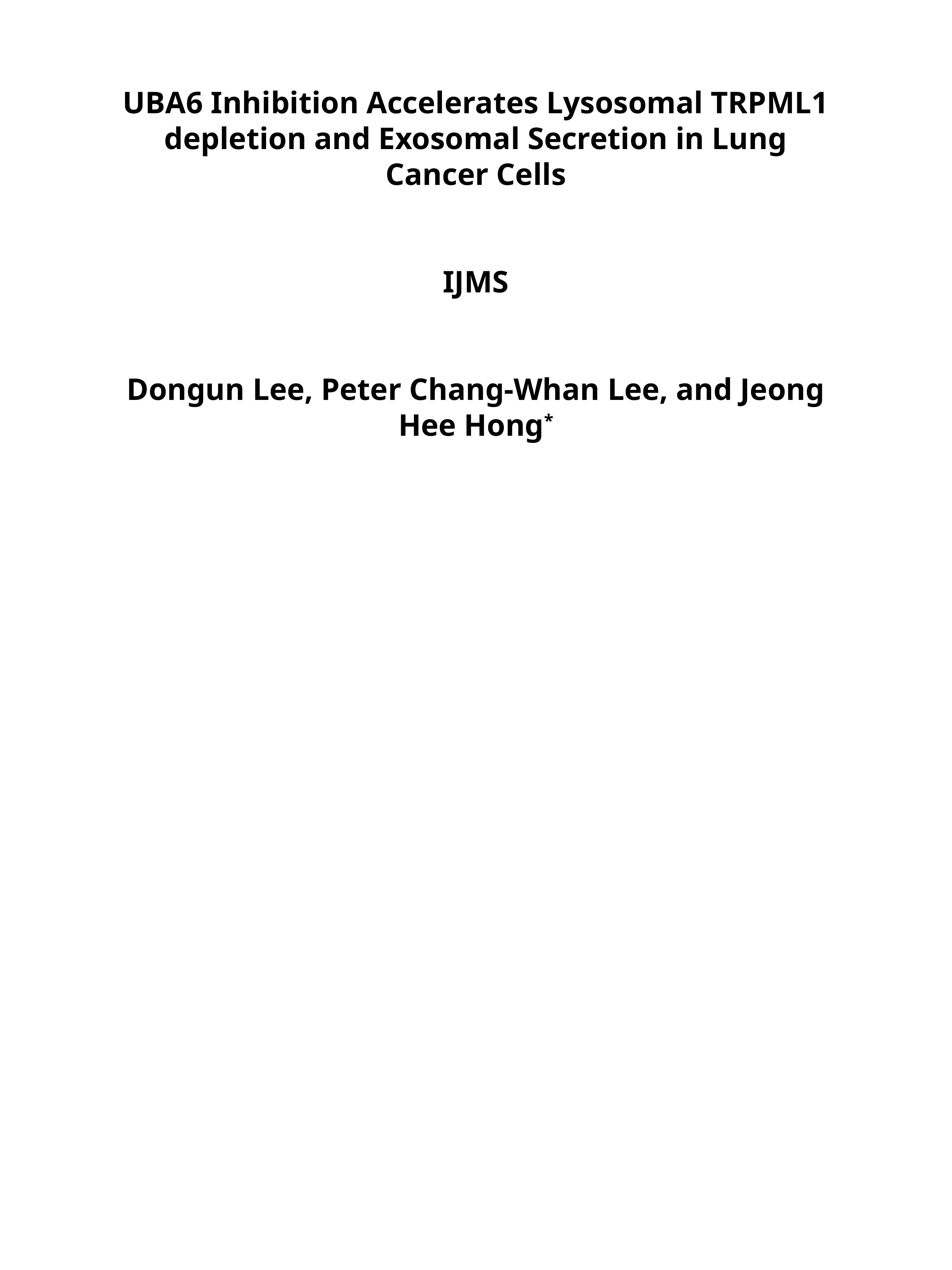

UBA6 Inhibition Accelerates Lysosomal TRPML1 depletion and Exosomal Secretion in Lung Cancer Cells
IJMS
Dongun Lee, Peter Chang-Whan Lee, and Jeong Hee Hong*

## Slide 2
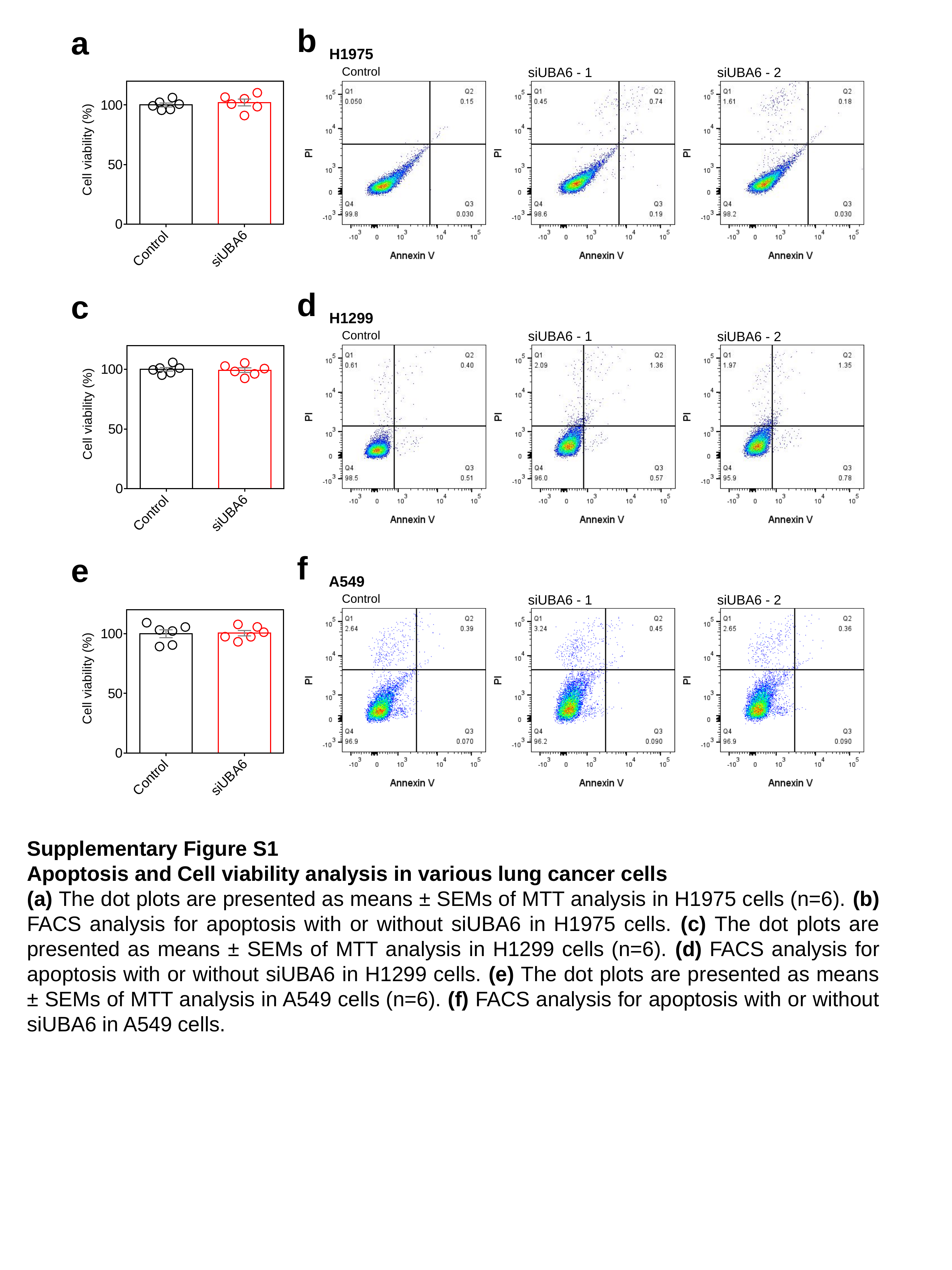

b
a
H1975
siUBA6 - 1
siUBA6 - 2
Control
d
c
H1299
Control
siUBA6 - 1
siUBA6 - 2
f
e
A549
Control
siUBA6 - 1
siUBA6 - 2
Supplementary Figure S1
Apoptosis and Cell viability analysis in various lung cancer cells
(a) The dot plots are presented as means ± SEMs of MTT analysis in H1975 cells (n=6). (b) FACS analysis for apoptosis with or without siUBA6 in H1975 cells. (c) The dot plots are presented as means ± SEMs of MTT analysis in H1299 cells (n=6). (d) FACS analysis for apoptosis with or without siUBA6 in H1299 cells. (e) The dot plots are presented as means ± SEMs of MTT analysis in A549 cells (n=6). (f) FACS analysis for apoptosis with or without siUBA6 in A549 cells.

## Slide 3
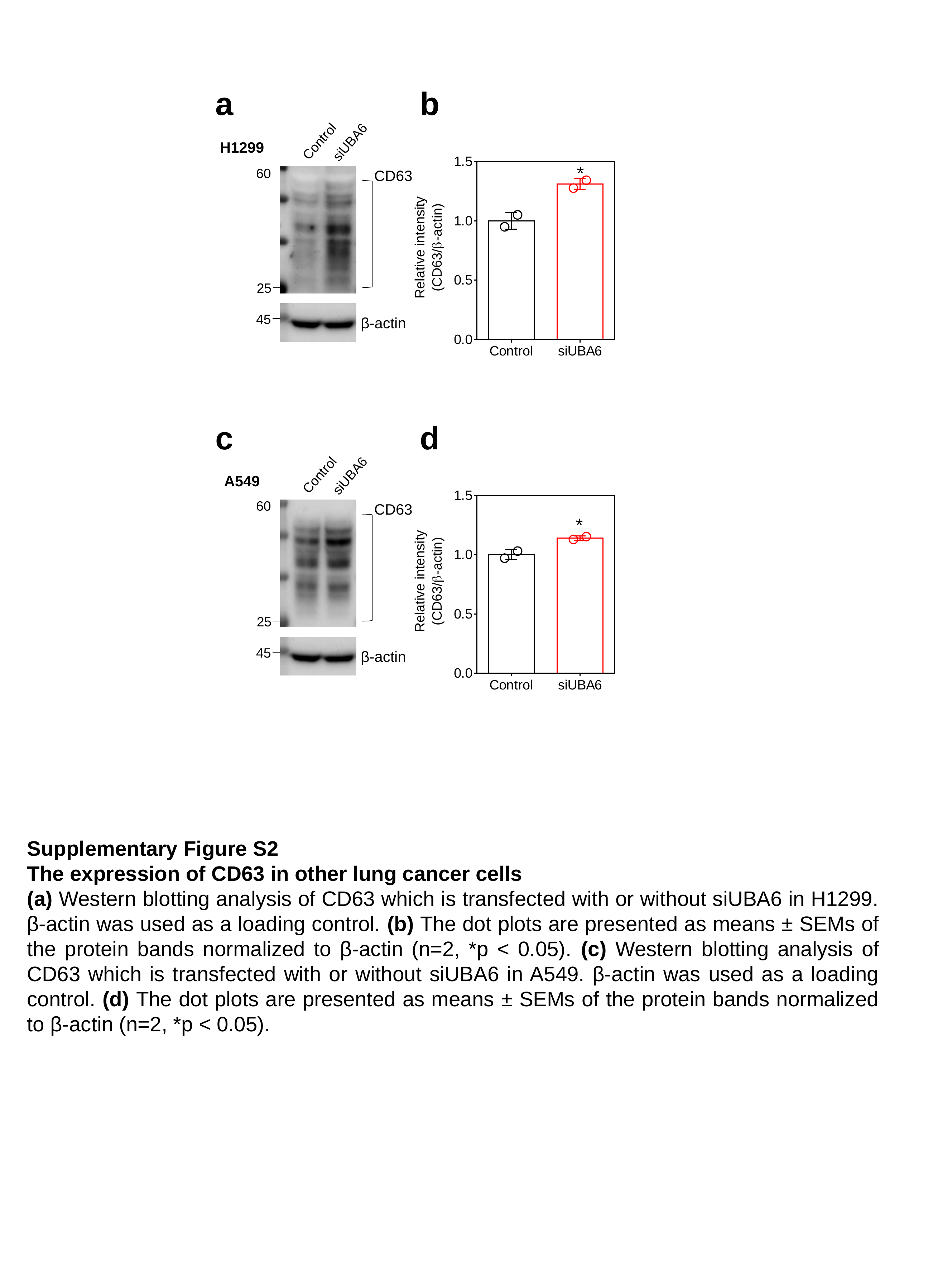

a
b
Control
siUBA6
H1299
60
CD63
25
45
β-actin
c
d
Control
siUBA6
A549
60
CD63
25
45
β-actin
Supplementary Figure S2
The expression of CD63 in other lung cancer cells
(a) Western blotting analysis of CD63 which is transfected with or without siUBA6 in H1299. β-actin was used as a loading control. (b) The dot plots are presented as means ± SEMs of the protein bands normalized to β-actin (n=2, *p < 0.05). (c) Western blotting analysis of CD63 which is transfected with or without siUBA6 in A549. β-actin was used as a loading control. (d) The dot plots are presented as means ± SEMs of the protein bands normalized to β-actin (n=2, *p < 0.05).

## Slide 4
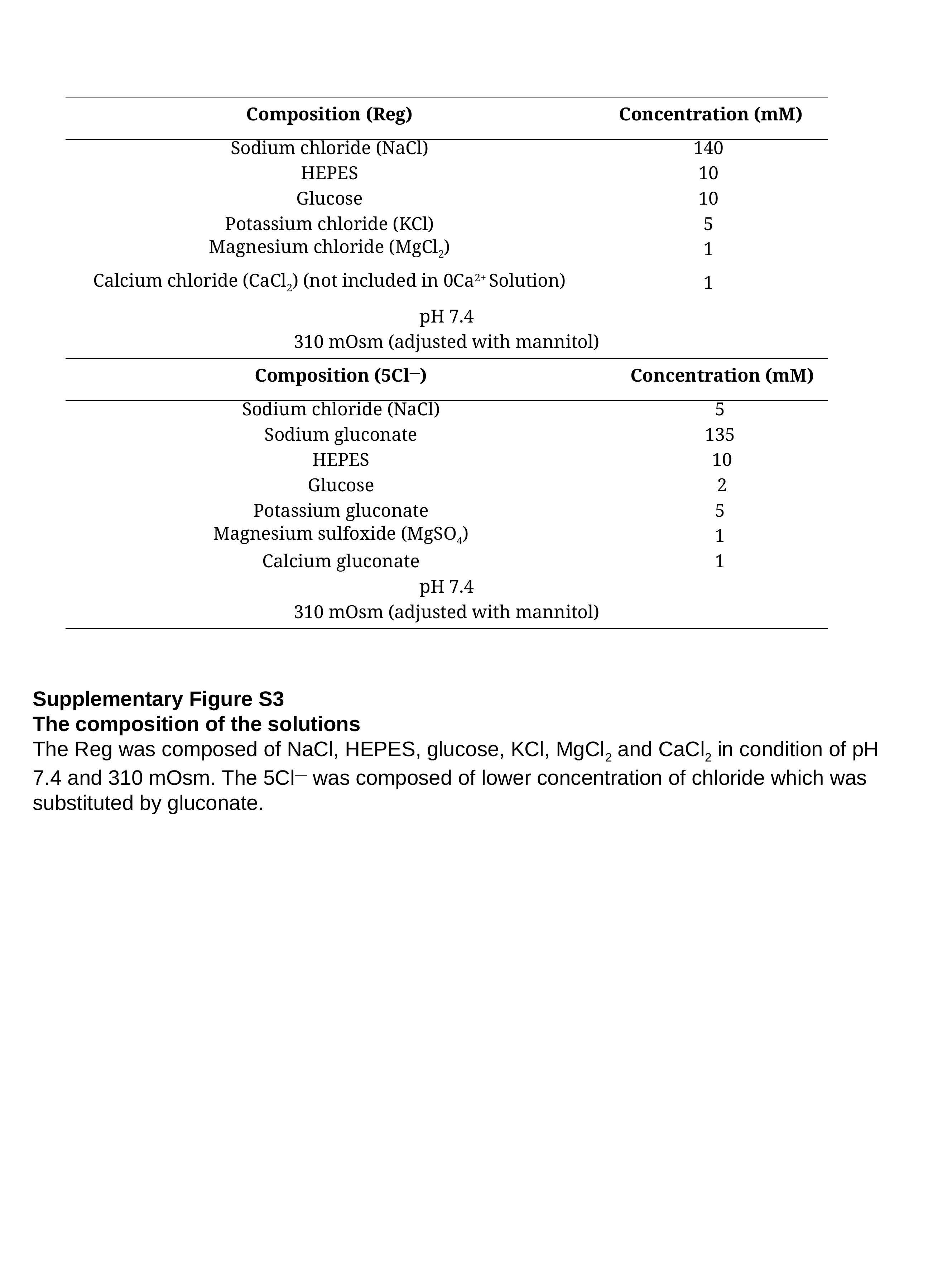

| Composition (Reg) | Concentration (mM) | |
| --- | --- | --- |
| Sodium chloride (NaCl) | 140 | |
| HEPES | 10 | |
| Glucose | 10 | |
| Potassium chloride (KCl) | 5 | |
| Magnesium chloride (MgCl2) | 1 | |
| Calcium chloride (CaCl2) (not included in 0Ca2+ Solution) | 1 | |
| pH 7.4 | | |
| 310 mOsm (adjusted with mannitol) | | |
| Composition (5Cl—) | | Concentration (mM) |
| Sodium chloride (NaCl) | | 5 |
| Sodium gluconate | | 135 |
| HEPES | | 10 |
| Glucose | | 2 |
| Potassium gluconate | | 5 |
| Magnesium sulfoxide (MgSO4) | | 1 |
| Calcium gluconate | | 1 |
| pH 7.4 | | |
| 310 mOsm (adjusted with mannitol) | | |
Supplementary Figure S3
The composition of the solutions
The Reg was composed of NaCl, HEPES, glucose, KCl, MgCl2 and CaCl2 in condition of pH 7.4 and 310 mOsm. The 5Cl— was composed of lower concentration of chloride which was substituted by gluconate.
